# GTax: improving de novo transcriptome assembly by removing foreign RNA contamination

**DOI:** 10.1186/s13059-023-03141-2

**Published:** 2024-01-08

**Authors:** Roberto Vera Alvarez, David Landsman

**Affiliations:** Computational Biology Branch, National Center for Biotechnology Information, Intramural Research Program, National Library of Medicine, NIH, Bethesda, MD USA

## Abstract

The cost and complexity of generating a complete reference genome means that many organisms lack an annotated reference. An alternative is to use a de novo reference transcriptome. This technology is cost-effective but is susceptible to off-target RNA contamination. In this manuscript, we present GTax, a taxonomy-structured database of genomic sequences that can be used with BLAST to detect and remove foreign contamination in RNA sequencing samples before assembly. In addition, we use a de novo transcriptome assembly of *Solanum lycopersicum* (tomato) to demonstrate that removing foreign contamination in sequencing samples reduces the number of assembled chimeric transcripts.

**Supplementary Information:**

The online version contains supplementary material available at 10.1186/s13059-023-03141-2.

## Introduction

Whole-genome and transcriptome sequencing has resulted in a greatly improved understanding of the biological complexities within organisms. Although whole-genome sequencing (WGS) is affordable for organisms with small genomes, it remains an expensive and complex task for organisms with larger genomes with more repetitive sequence regions [1]. Nevertheless, whole-transcriptome sequencing (WTS), also known as RNA sequencing (RNA-Seq), is a cost-effective means [2] to study differential gene expression profiles [3, 4], phylogenomics [5, 6], or plant evolution [7, 8]. It is particularly useful to create suitable reference transcriptomes for unannotated organisms using computational approaches called de novo transcriptome assemblies [9]. Assembled transcripts are annotated through the identification of homologous genes, proteins, and functional domains that could be cross-referenced with other public databases, such as Gene Ontology (GO) [10].

The lack of a reference genome of several species that have significant public health, economic, and environmental importance is a barrier in many studies. For example, green plants (*Viridiplantae* kingdom) or corals (*Anthozoa* class) are important groups of organisms but have limited annotations in public databases. The National Center for Biotechnology Information (NCBI) Genome database [11] contains 379 annotated reference genomes for the *Viridiplantae* kingdom but only 17 for the *Anthozoa* class. The NCBI Taxonomy database, however, shows 262,944 taxa in the *Viridiplantae* kingdom [12] and 9651 taxa in the *Anthozoa* class [13] (as of November 9, 2023).

RNA-Seq contamination has played an important role in misleading multiple research conclusions (see the “Background” subsection). Such contamination is more troublesome when the target organism does not have a reference genome with annotation available in public databases. In this manuscript, we discuss the effects of RNA-Seq contamination on de novo transcriptome assembly that uses the Trinity pipeline. We evaluate the quality of seven de novo transcriptome assemblies for *Solanum lycopersicum* (tomato). In addition, we present GTax, a taxonomic structured database of genomic sequences that can be used with BLAST [14] or other tools like Kraken2 [15], for taxonomic classification and filtering. GTax database can be created using a public python package as described in https://gtax.readthedocs.io/. This approach allows the use of BLAST for efficiently detecting and eliminating contaminant reads in RNA-Seq data.

As we mentioned before, seven transcriptomes were de novo assembled from simulated and real RNA-Seq sequencing data to evaluate the effect of RNA-Seq contamination on the quality and completeness of the assemblies. The first transcriptome was assembled using randomly selected 100-bp long reads extracted from the tomato reference transcriptome. The second transcriptome was assembled by adding to the previous reads two million contaminant reads (20%) randomly selected from eight GTax taxonomy groups. Finally, the last seven transcriptomes were assembled from 10 tomato wild-type RNA-Seq samples with different contamination levels.

BLAST is recognized as the most sensitive tool for sequence similarity searches but its use for taxonomic classification of short RNA-Seq reads is limited due to database sizes and computational resources. As mentioned previously, we investigated the effect that RNA-Seq contamination has on de novo transcriptome assembly. We used BLAST as sequence aligner, to achieve the highest-quality results. GTax is designed as a practical sequence database that will allow us to use BLAST for taxonomic classification of short RNA-Seq without losing genomic information available in the NT database. Similarly, Kraken2 is also used with GTax to remove contamination and the two resulting de novo assembled transcriptomes are compared.

Final assemblies were compared to the reference genome and transcriptome using Benchmarking Universal Single-Copy Orthologs (BUSCO) and rnaQuast.

### Background

There are three main companies that provide RNA-Seq technologies: Illumina, Oxford Nanopore Technologies (ONT), and Pacific BioSciences (PacBio). Illumina remains the dominant RNA-Seq platform, as the company’s technology reports extremely low error rates and is affordable for high-sequence coverage depths [16]. Illumina-generated RNA-Seq short reads, however, can produce artifactual chimeras and fragmented transcripts during de novo transcriptome assembly [1]. ONT and PacBio sequencing technologies are designed to generate RNA-Seq long reads that could be used to sequence full-length transcripts. Nevertheless, these technologies result in higher error rates and lower throughput. Alternative approaches have been developed using hybrid de novo transcriptome assembly, including both short and long RNA-Seq reads. These methods improve the quality of the assembly [17], but the cost of using multiple sequencing platforms is a limitation of its general applicability [18].

Multiple assemblers (e.g., Trinity [19], Trans-ABySS [20], SPAdes [21]) process Illumina RNA-Seq short reads. Trinity is the most commonly used tool for de novo transcriptome assembly. It was developed specifically for transcriptome assembly and uses de Bruijn graphs to generate multiple isoforms of a gene. In addition, Trinity offers an in silico normalization method to process samples with different sequencing depths. Holzer and Marz compared assemblers and reported that these tools outperformed others; no tool, however, delivered perfect results for all analyzed data sets [2].

As noted above, RNA-Seq short read-based assemblers generate transcriptomes with fragmented or chimeric isoforms. Therefore, additional steps are needed to identify spurious transcripts and assess the quality of assemblies. Multiple approaches have been developed to reduce false-positive transcripts assembled de novo; however, most of them require the availability of a closely related reference genome for the target organism [2]. Transcript abundance is quantified by calculating transcripts per million (TPM) as described in [22]. The low-expression contigs with TPM levels that are lower than a cutoff value are discarded. NCBI BLAST tools are used to identify spurious transcripts by sequence homology searches against multiple databases. BUSCO [23, 24] and rnaQuast [25] can be used to evaluate the quality of the assembly. BUSCO estimates the completeness and redundancy of an assembly based on universal single-copy orthologs. rnaQuast is intended for testing different assembly methods and pipelines on well-known organisms.

Theoretically, assemblers expect RNA-Seq data from a single organism. Therefore, the quality of a de novo assembly depends not only on the computational pipeline but also on the quality and purity of the RNA-Seq data. Contamination, however, is more common than expected in RNA-Seq samples [26, 27]; it is particularly problematic in samples from organisms for which there is no reference genome by which to frame the analysis [28]. Contamination of genomic and transcriptomic data can be classified as foreign and cognate. Foreign RNA-Seq contamination involves reads that originate from off-target, contaminant organisms, and cognate are reads that originate from off-target RNA species [9].

Contamination has been the source of many inaccurate findings (e.g., a report of high rate of horizontal gene transfer (HGT) found in the tardigrade genome [29]). In this case, HGT was later rejected by Koutsovoulos et al. after finding bacterial contamination in the data [30]. Downstream analysis, such as the inference of phylogenomic trees, produces wrong classifications of taxonomies [31]. Inaccurate assemblies create bias in the analysis of a non-dietary origin of exogenous plant miRNAs reported to cross the mammalian gastrointestinal track [32]. Further, a common assumption is that contamination is *not* a problem when a reference genome exists [33]; however, many studies have demonstrated that reference genomes should be used prudently due to existing contamination in public databases [8, 27, 34, 35].

Detecting and removing contamination from WGS or WTS data prior to the assembly is a critical step in a pipeline for de novo transcriptome assembly. Ballenghien et al. stated that “Bioinformatic pipelines for NGS-based population genomic data should be further developed/improved in order to account for the probable existence of between-species and within-species contamination” [28]. We address this important issue here and demonstrate how contamination can bias a de novo assembled transcriptome.

Detecting the contamination in RNA-Seq short reads is complex due to sequence similarity between genes in distant taxonomic species. An illustrative example is the photosynthesis-related genes found in genomes of phototrophic bacteria that originate from plants [36]. Horizontal Gene Transfer (HGT) events are also challenging examples of the complexity of detecting contamination on RNA-Seq data.

BLAST tools can be used to align RNA-Seq short reads to public databases of sequences to associate reads with one or more taxonomies. This is time- and resource-consuming, however, even when using modern cloud-computing platforms [37]. K-mer-based methods have been developed to accelerate the computation but at the cost of reducing the sensitivity of the taxonomy classification. Although k-mer-based tools are reported to be 900 times faster than BLAST, the latter is a more sensitive tool for sequence similarity identification [38]. Kraken2 [15], a k-mer-based tool, can be used for detecting contamination in RNA-Seq short reads through taxonomy classification. CLARK [39] is another tool that uses a supervised sequence classification with discriminative k-mers. CONSULT [40] tests whether k-mers from a query fall within a user-specified distance of the reference dataset using locality-sensitive hashing. Kaiju [41] executes a taxonomic classification for high-throughput sequencing reads but cannot be used to extract contaminant reads from raw sequence files. Conterminator [27] detects contamination in nucleotide and protein sequence sets using an all-against-all sequence comparison. Other tools for detecting RNA-Seq data contamination are FastQ Screen [42], which uses Bowtie and BWA but is limited by the existence of a reference genome, and RNA-QC-Chain [43], which can remove rRNA reads and identify foreign species in the sample using Hidden Markov Model searches but is incapable of identifying and removing foreign contaminant reads. For a detailed comparison among these tools, see Ounit et al. [39] and Cornet [44].

Public databases at host institutes of the International Nucleotide Sequence Database Collaboration include a taxonomic classification for genome and transcriptome deposited data. The NCBI Sequence Read Archive [45] (SRA) uses an *in-house* developed taxonomic classification tool named Sequence Taxonomic Analysis Tool (STAT) [46]. STAT is a scalable k-mer-based tool for fast assessment of taxonomic diversity intrinsic to SRA submissions. Although it offers valuable information and metadata, it was not designed for distribution. Downloading raw reads filtered by selected taxonomic identifiers from SRA archives is not possible.

## Results and discussion

### GTax, a taxonomic structured database of genomic sequences

The identification of contaminant reads in RNA-Seq samples is complex, especially when the source of contamination is unknown. It is also limited to the genomic information deposited in public databases. The use of traditional sequence similarity search tools, such as BLAST, is inefficient in identifying contamination in RNA-Seq raw data files, which may contain from 10 to 100 million reads. Public BLAST databases, such as NT and NR, have grown to more than 400 GB of compressed indexes, making the screening of millions of short sequences impractical. As of November 9, 2023, the NT database contained 100,293,765 sequences and 1,375,728,060,136 total bases (UNIX command: blastdbcmd -info -db nt), and the NR database contained 631,584,287 sequences and 247,294,550,504 total residues (UNIX command: blastdbcmd -info -db nr).

NCBI released a new tool, Datasets [47], that gathers data from across NCBI databases using command line instructions. This tool can be used to query the NCBI Genome databases and retrieve all available assemblies. It also provides machine-readable metadata that can be used for classification and filtering. We used Datasets metadata for the creation of GTax, a taxonomic structured database of genomic sequences that includes a subset of RefSeq reference genomes, if available, or the latest assembly (Additional file 1). Sequences were filtered by RefSeq Accession prefixes [48] to reduce redundancy and possibly contaminated sequences (see the “Methods” section for details). The sequences were organized into 19 mutually exclusive and hierarchical taxonomic groups; see Table 1. For example, taxonomies in the *Viridiplantae* kingdom are divided into three GTax groups, *Liliopsida* includes all monocotyledon sequences, the Eudicotyledons group includes all dicotyledon sequences, and other taxa in the *Viridiplantae* kingdom not in these two groups are placed in the *Viridiplantae* group at a higher level. The same principle is applied to the *Chordata* phylum and all taxonomy groups from *Neoteleostei* to *Sarcopterygii*. Finally, all remaining *Eukaryote* taxa are placed in the Eukaryota taxonomy group.

**Table 1:** GTax database content

Taxonomy group	No. of taxonomies	RefSeq sequences	Size (GB)
Bacteria	8558	16,137	35.11
Archaea	336	554	0.90
Liliopsida	21	265	26.83
Eudicotyledons	79	880	39.17
Viridiplantae	12	184	4.52
Fungi	82	797	1.70
Arthropoda	101	1364	26.06
Neoteleostei	75	1437	44.60
Actinopterygii	37	1047	45.52
Glires	25	2178	30.44
Primates	20	433	39.32
Carnivora	31	286	28.89
Artiodactyla	28	447	36.90
Amphibia	9	122	30.36
Sauropsida	61	1073	46.17
Sarcopterygii	29	229	26.83
Chordata	11	301	13.99
Eukaryota	71	803	8.76
Viruses	11,071	13,555	0.44

This taxonomic structured division of the genomic sequences in GTax keeps phylogenetically closely related species in the same taxonomy group and significantly reduces the size of the searchable BLAST database. The *Sauropsida* group, which is the biggest group, contains 1073 sequences and 46,172,754,879 total bases, only 6.84% of the NT database.

The GTax database is substantially smaller than the NCBI NT database without a loss of any genomic information. The database reduces sequence redundancy by selecting the biggest available genomic sequences for each organism. For example, when an organism has complete chromosomes, there is no need to include any other sequences for that organism as all RNA-Seq reads from that organism will align to the chromosomes. Sequences from organisms with incomplete genomes are included using all contigs or scaffolds available in the public databases. In these cases, other sequences generated from those contigs or scaffolds are also included in GTax. In our opinion, using larger databases like NCBI NT with k-mer-based tools like Kraken2 will not be different from using the same tool with GTax. The utility of Gtax is not to identify complete sequences like transcripts or mRNA that are present in public databases. Rather, the aim is to classify short RNA-Seq reads to the appropriate organism. Therefore, single copies of chromosomes, contigs, or scaffolds are sufficient for this specific task. Finally, run time and computer resources are reduced using GTax which also applies to all k-mer taxonomic classification systems, including Kraken2.

Our taxonomic classification workflow uses a two-step approach to detect contamination. In the first step, all reads are screened against the target organism’s GTax taxonomy group. Here, we assume that RNA-Seq reads from a target organism will be aligned to the correct taxonomy group when the target organism has a reference genome or an assembly at any level included in GTax; these reads are identified as “correct” reads. If there are no sequences for the target organism in GTax, but genomic sequences from a phylogenetically closely related species are present, then some percent of the reads will align with the correct taxonomy group. In the second step, reads which did not align with a sequence in the organism’s GTax taxonomy group are screened against the rest of GTax taxonomy groups to identify contaminant reads.

Finally, if the target organism has neither genomic sequences nor phylogenetically closely related sequences from species in GTax, reads will be classified as unidentified after screening the rest of the GTax taxonomy groups in the second step.

Contaminant reads from organisms with genomic sequences in GTax align with their respective taxonomy groups. Those reads are identified as contamination and marked for removal. This approach identifies contaminant reads for known organisms with sequences available in GTax. Most of the common contaminants, such as bacteria, fungi, or human, can be identified using the GTax taxonomy groups. This approach, however, will not identify contaminant reads when information about the source is not available in public databases.

Our approach is initiated by screening using BLAST searches of the RNA-Seq reads against the taxonomy group of the target organism. Running time will depend on the number of reads to screen and the taxonomy group used as BLAST database. The most time-consuming case is when the target organism belongs to the *Sauropsida* clade, the biggest group in GTax. In this case, we screen millions of RNA-Seq reads against less than 6% of the NT database which is 15 GB of BLAST indexes. BLAST, using 16 threads (-num_threads 16), requires 16 GB of RAM for processing on average 303 reads per minute using an AMD EPYC 7543 CPU.

We tested our approach in two ways. First, we selected 15 organisms from 12 taxonomy groups with reference genomes included in the GTax database. We generated overlapping single-end reads, 100 bp long, sequentially from the reference transcripts with an overlapping window of 50 bp. For the two *Bacteria* included, the reads were generated from the reference genome. Second, 15 RNA-Seq samples were selected from the SRA database for organisms without a reference genome, in addition to a highly contaminated human sample and a WGS sample from *Pseudomonas fluorescens*.

Tables 2 and 3 show the groups of “generated 100 bp overlapped reads” from organisms with reference genomes. The correct taxonomy group for each organism is identified with a red background.

**Table 2:**
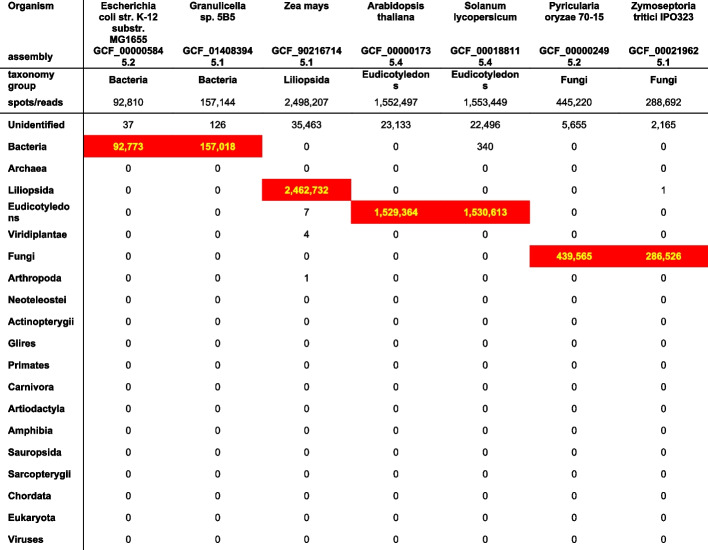
Alignment with each GTax taxonomic group of 100-bp overlapped sequences generated from the reference transcriptome of several organisms. Reads aligned to the target organism’s GTax taxonomic group are shown in red

**Table 3:**
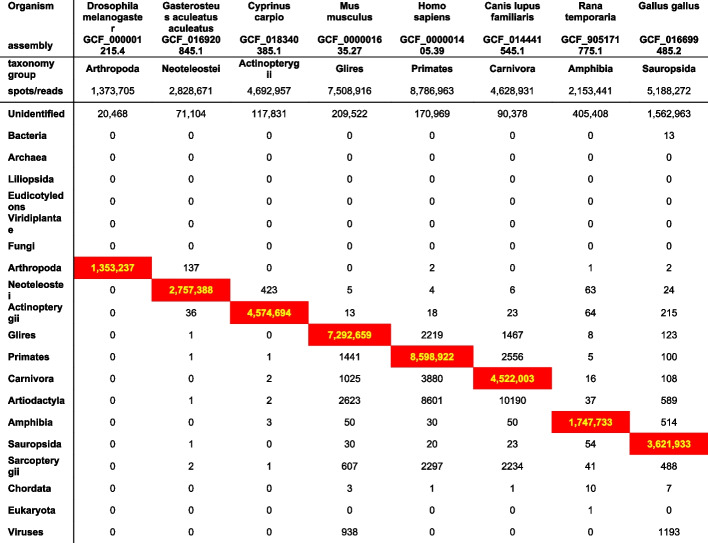
Alignment with each GTax taxonomic group of 100-bp overlapped sequences generated from the reference transcriptome of each organism. Reads aligned to the target organism’s GTax taxonomic group are shown in red

Table 2 includes the first group: *Bacteria*, *Green Plants*, and *Fungi*. More than 98% of the reads from these organisms are aligned with the correct taxonomy group. In the specific case of *Solanum lycopersicum* (tomato), 340 reads are aligned with the Bacteria taxonomy group, which may indicate some level of contamination in the transcriptome.

The second group of organisms, presented in Table 3, is different. Although more than 97% of the generated reads align with the correct taxonomy group for all organisms except *Rana temporaria* (frog) and *Gallus gallus* (chicken), there is an increased number of reads aligned with other taxonomy groups, indicating a varied amount of contamination on their reference genomes. The frog and chicken examples show a lower number of reads aligned with the correct taxonomy group but also few reads aligned with other groups. We suspect that, in addition to some contamination, these assembled transcriptomes include some chimeric transcripts that are not aligned with any genomic sequence. Chimeric transcripts also are the most probable explanation for the small percentage of generated reads that remain unidentified in all examples. After further investigation, we confirmed that all unidentified reads belong to computationally predicted transcripts (accessions prefixes with XR_ and XM_) included in the transcriptomes and are probably not valid (for more details on RefSeq prefixes, see NCBI RefSeq Accession prefixes [48]). These experiments demonstrate that “correct” reads can be identified in high numbers when the target organism has a reference genome, or an assembly, included in GTax.

Tables 4 and 5 provide the results for the instances when the target organism sequences are not included in GTax. These tables include a row in yellow with the percentage of filtered reads that can be used to assemble the transcriptome after the removal of contaminant sequences. Cells colored with the same background, the correct taxonomy group, and the remaining unidentified reads for each organism (columns) are summed to generate the “Percentage of reads for assembly” row (yellow in all organisms).

**Table 4:**
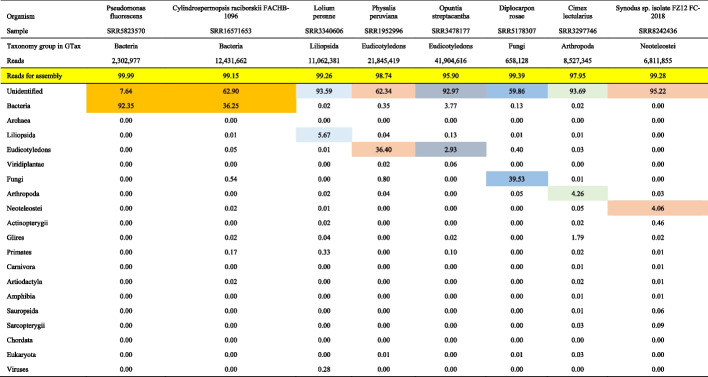
Samples from SRA database for organisms without a reference genome

Values are expressed in percentages. Reads in cells of the same color for each sample are summed to generate the final reads to assembly

**Table 5:**
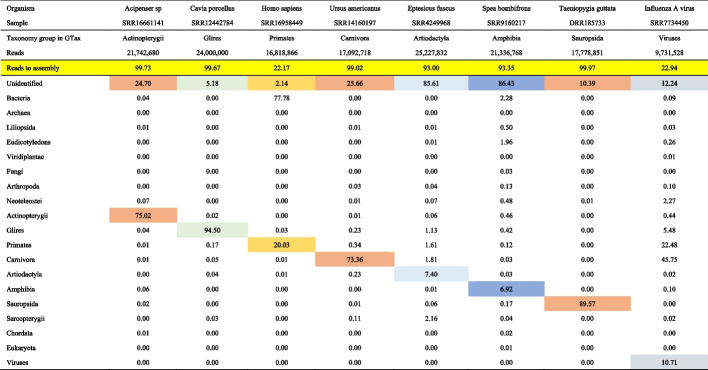
Samples from SRA database for organisms without a reference genome

Values are expressed in percentages. Reads in cells of the same color for each sample are summed to generate the final reads to assembly

Table 4 shows low levels of contamination for the RNA-Seq samples. For *Pseudomonas fluorescens* (SRR5823570), which has phylogenetically closely related species in GTax, 92.35% of the reads aligned with the correct taxonomy group, with 7.64% unidentified reads (these reads sum to 99.99% of the reads, are labeled as “correct,” and are used in the assembly step). In addition, 36.25% of reads from the other bacterium, *Cylindrospermopsis raciborskii FACHB-1096* (SRR16571653), are aligned with the correct taxonomy group, while 62.90% remain unidentified. Both *Fungi* and *Primates* sequences contaminate this sample. Overall, 99.15% of the reads can be used for assembly. Most of the reads for the three plant examples are unidentified, with some contamination detected from *Bacteria*, *Fungi*, and *Primates*. The Eudicotyledons examples, *Physalis peruviana* and *Opuntia streptacantha*, display a different level of reads aligned with the correct taxonomy group. For *Physalis peruviana*, which is closely related to tomato, 36.40% of the reads align with the Eudicotyledons taxonomy group, whereas *Opuntia streptacantha* does not have a closely related organism in the database, and most of the reads are unidentified (92.97%).

Table 5 shows the second group of analyzed samples. Similarly, organisms such as *Synodus sp. isolate FZ12 FC-2018* with a low number of reads aligned with the correct taxonomy group (Table 4) and *Cavia porcellus*, closely related to mouse, have 94.50% of reads aligned with the correct taxonomy group (Table 5).

The human sample included in this example (SRR16958449) contains 20.03% of the reads aligned with *Primates* and 2.14% of reads unidentified, for a total of 22.17% of the reads ready for assembly. This sample, however, contains a high level of bacterial contamination, 77.78% of the reads. The SRA STAT report also similarly shows 78.45% of contaminated reads (see the “Analysis tab” at https://trace.ncbi.nlm.nih.gov/Traces/sra/?run=SRR16958449). This sample was extracted from vascular aortic smooth muscle cells, and no bacterial presence was reported in the study. All other samples in this study show similar bacterial contamination. The general approach includes the unaligned reads for further analysis. In cases with well-annotated reference genomes, such as human, mouse, yeast, or some bacteria, however, we recommend using reads aligned only with the target taxonomy group.

The *Influenza A virus* sample included in Table 5 displays a high number of reads aligned with the *Primates* and *Carnivora* taxonomy groups. Virus samples are usually collected from host organisms. In this case, for *Mustela putorius furo* (domestic ferret), which belongs to the *Carnivora* taxonomy group, 22.48% of the reads in these samples align with *Primates*, indicating significant human contamination.

SRA samples analyzed in Tables 4 and 5 were also decontaminated with Kraken2 and GTax as shown in supplementary Tables S3A and S3B (Additional file 2 Tables S3A and S3B). Kraken2 classifies more reads than BLAST in the target organism’s GTax taxonomy group. Also, Kraken2 classifies more reads into other taxonomy groups reducing the number of “unidentified” reads. These are expected results as Kraken2 uses a k-mer-based algorithm with maximum k-mer length of 35 which is much smaller than our sequence read lengths (≥ 100 bp). Local alignments of 35 bp can occur but this only represents 35% of the query read if the read is 100 bp or less than that for longer reads. Conversely, BLAST options were modified to report alignments with query coverage larger than 75% of the read; thus, BLAST-based alignments are more sensitive than those reported by Kraken2. Nevertheless, Kraken2 results are very similar to BLAST.

As mentioned previously, HGT events are challenging and interesting situations that need extra care when removing foreign RNA-Seq contamination. Our algorithm uses a two-step approach to detect contamination. In the first step, all reads are screened for the target organism’s GTax taxonomy group. Reads aligned to that group of sequences will be marked as non-contaminant. In the second step, unidentified reads are screened against the rest of GTax taxonomy groups. Identifying reads as non-contaminant in the case of HGT in the target species will depend on the availability of a reference genome or the presence of similar HGT in other phylogenetically close species (species in the same GTax taxonomy group). There are three situations that we can visualize in the case of HGT events. First, if the target organism reference genome is in GTax, all reads, including the HGT reads, that align to the target organism’s GTax taxonomy group sequences will be no contaminant. Those reads will not be screened against the other GTax taxonomy groups. Second, if no reference genome is available but the HGT events are common in other phylogenetically close species in the target organism’s GTax taxonomy group, then, those HGT reads will be aligned to the target organism’s GTax taxonomy group sequences and will be considered non-contaminant. Finally, if the HGT reads cannot be aligned to any of the sequences included in the target organism’s GTax taxonomy group, then our algorithm will fail, and assign those reads as contaminants. These would be considered rare cases.

Detecting RNA-Seq contamination is limited to the sequences included in the database and the confidence that those sequences are free of contaminants. The algorithm described in this manuscript identifies “known” contaminants and suggests using “unidentified” reads for assemblies with the assumption that in these reads could remain contaminants. Post-assembly quality control tools like CoCro [49] could be used to detect possible remaining contaminations after the assembly is completed.

We have developed a python package that can be used to generate the GTax taxonomy group FASTA files for the creation of BLAST indexes (https://gtax.readthedocs.io/).

### Effect of RNA-Seq contamination on de novo transcriptome assembly

We used the *Solanum lycopersicum* (tomato) reference transcriptome and a wild-type tomato RNA-Seq dataset to study the effect of foreign contamination on de novo transcriptome assembly. The tomato genome is well annotated with a reference genome and transcriptome available (assembly ID GCF_000188115.4). The current annotated genome includes 45,901 transcripts (see https://www.ncbi.nlm.nih.gov/genome/?term=txid4081[orgn]).

Seven tomato transcriptomes were assembled de novo using different pre-processing approaches. The first transcriptome assembly contains randomly extracted 100-bp long reads from the tomato reference transcriptome. This was repeated four times to produce four paired-end samples of ~9 million reads each. The second transcriptome was assembled with the same tomato reads as the first plus two million (~20%) randomly selected contaminant reads added to each sample (4% *Bacteria*, 1% *Archaea*, 10% *Fungi*, 1% *Arthropoda*, 1% *Chordata*, 1% *Metazoan*, 1% *Eukaryote*, and 1% *Viruses*). We added more *Fungi* and *Bacteria*, as they are the most probable plant sample contaminants. It is important to note that these two sets of generated samples contain the same tomato reads. The only difference is the contaminant reads added to the second set. No decontamination was executed on these reads as we aim to quantify the effect of the contamination added to the second set of samples to the final assembled transcriptome.

**Fig. 1:**
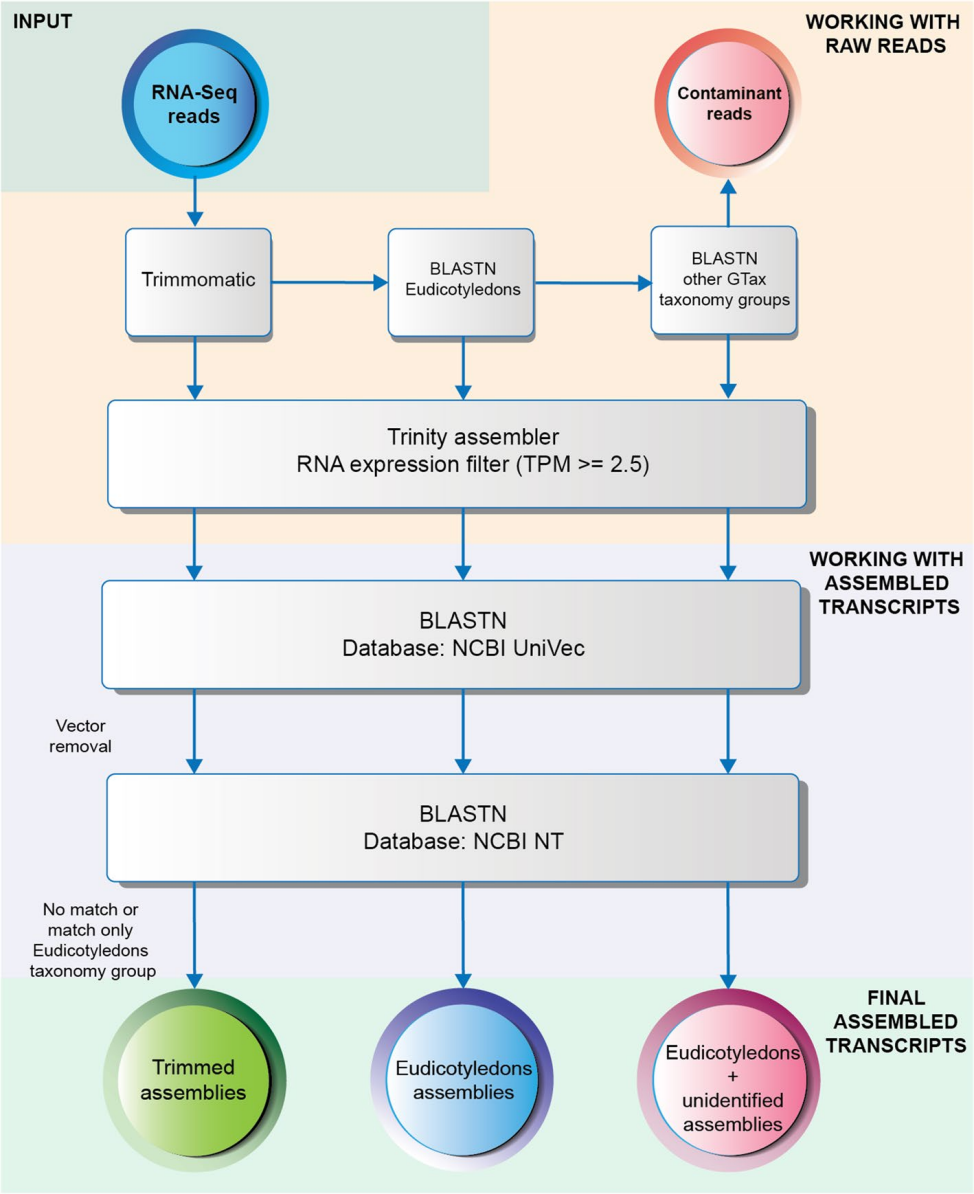
Workflow to remove vectors and contaminated transcripts after assembly completion. Different levels of decontamination of the SRA samples were used to assemble three transcriptomes: Trimmed, Eudicotyledons, and Eudicotyledons + unidentified

The five other transcriptomes were assembled from ten tomato wild-type RNA-Seq samples selected from the SRA database (Additional file 3, tab: “Table 1 – Tomato WT samples”). The samples belong to four different BioProjects from different plant tissues. The first RNA-Seq transcriptome was assembled after trimming the adapters and filtering out low-quality reads (Trimmed assembly in Fig. 1). The second and third RNA-Seq transcriptomes were assembled with trimmed reads that match the Eudicotyledon taxonomy group in Gtax aligned with BLAST and Kraken2 respectively (*Eudicotyledons* assembly in Fig. 1). The fourth and fifth RNA-Seq transcriptomes were assembled with the Eudicotyledons-matched reads plus the unidentified reads that remain after screening the samples against all other Gtax taxonomy groups aligned with BLAST and Kraken2 respectively (Eudicotyledons + unidentified assembly in Fig. 1).

After assembly, transcriptomes were filtered to remove lowly expressed transcripts using a TPM cutoff of ≥ 2.5. Two different post-assembly decontamination screenings were performed. First, transcripts were screened with BLASTN against the NCBI UniVec database [50] to detect and remove vectors. Then, a second BLASTN screen is done against the NT database to identify and remove contaminated transcripts. This final BLAST screen is the starting point of the annotation process [37].

**Fig. 2:**
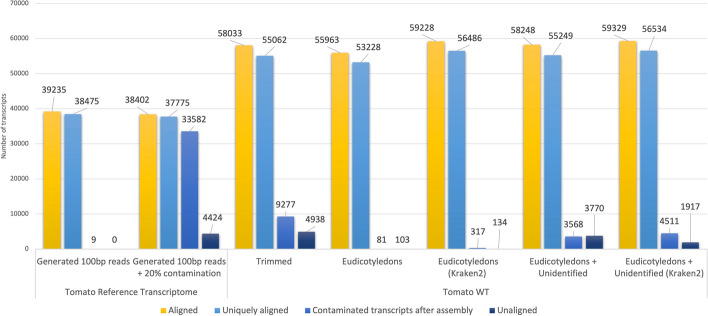
Alignment results reported by rnaQuast for seven tomato transcriptomes de novo assembled in this study (Supporting data for Fig. 2 is in Additional file 3)

To evaluate the quality of RNA-Seq assemblies, we used rnaQuast software [25]. Figure 2 shows the rnaQuast results of aligned transcripts for the seven assemblies. The transcriptome assembled with the 100-bp generated reads from the tomato reference transcriptome contains 39,235 transcripts aligned with the reference genome (38,475 uniquely aligned). Although this assembly used reads generated from only the tomato reference transcriptome, 9 contaminant transcripts were identified, using BLASTN against the NT database, and removed after assembly. As expected, these 9 transcripts aligned with *Bacteria* sequences (in Gtax), supporting the assumption that bacterial contamination is minimally present in the tomato reference transcriptome, as identified in Table 2. No transcripts in the transcriptome were unaligned with the reference genome after all the post-processing steps. Transcripts identified in Fig. 2 as unaligned are considered false-positive transcripts (probably chimeric) and remain in the transcriptome as there are no available methods to detect them accurately. Thus, Trinity does not generate chimeric transcripts when using only reads generated from the tomato reference transcriptome.

The second transcriptome assembled shows a different distribution of transcripts. This transcriptome was assembled with the same tomato reads mentioned before plus 20% contaminant reads from several other species. In this case, 833 fewer assembled transcripts align with the reference genome. Post-assembly decontamination steps remove 33,582 contaminated transcripts, but 4424 unaligned transcripts remain in the transcriptome. These transcripts are not detected by any of the post-processing steps. They incorrectly remain in the assembled transcriptome as valid transcripts. Trinity seems to mix some reads from the contaminant sequences with tomato sequences creating chimeric transcripts absent in the previous assembly. The 4424 additional transcripts and the reduction of aligned transcripts from 39,235 (transcriptome with only tomato reads) to 38,402 (transcriptome with tomato and contaminant reads) demonstrate how RNA-Seq contamination affects the quality and completeness of the final assembled transcriptome.

The last five transcriptomes using tomato wild-type RNA-Seq data corroborate the assumption that contamination affects the final assembly significantly. Transcripts assembled from the trimmed reads contain 4938 unaligned transcripts that are reduced to 103 when only Eudicotyledons reads are used. This number increases to 3770 when the unidentified reads are added to the samples for BLAST-based alignments. The Eudicotyledons assembly, however, includes 2070 fewer transcripts aligned with the reference genome than does the trimmed assembly and 2285 fewer than the Eudicotyledons + unidentified assembly.

Kraken2-based assemblies show slightly different numbers. For the Eudicotyledons, more transcripts are aligned to the reference genome than those generated by BLAST but also more unaligned transcripts are produced.

rnaQuast reports an increase in the duplication ratio from 1.2 in the first two assemblies to 1.7 in the five others, producing a difference of > 16,000 transcripts (see Additional file 3, tab: “Table 2 – rnaQuast short report”). There seems to be a proportional relationship between duplicated transcripts and the number of reads used in the assembly.

**Fig. 3:**
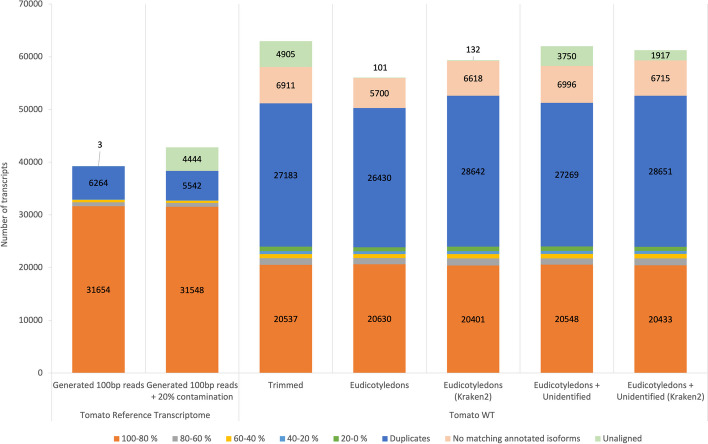
Percentage of sequence coverage of annotated isoforms is reported in color for each assembly (supporting data for Fig. 3 is in Additional file 3)

We also assessed the exon coverage of the alignment of each assembled transcript set to the tomato genome. BLASTN was used to align the assembled transcripts to the tomato reference genome. BLAST high-scoring segment pairs (HSPs) were used to quantify the sequence coverage of the annotated transcripts by counting the overlap between HSPs and annotated exons. Figure 3 shows the number of transcripts that match annotated isoforms in the tomato reference genome. In all cases, there is a reduction in the number of transcripts that match annotated isoforms when contamination is present. Using the generated 100-bp tomato reads, Trinity assembled 68.96% of annotated transcripts with more than 80% sequence coverage. This is reduced to 44.94% using the Eudicotyledons reads (BLAST), which is the best coverage obtained with the SRA samples. The figure shows that although Kraken2-based assemblies produce more transcripts that are aligned to the genome as described in Fig. 1, there are less transcripts that match the annotated isoforms with more than 80% sequence coverage, a higher number of duplicated transcripts, and a higher number of transcripts that do not match any annotated isoform. This demonstrates that a slight modification in the sensitivity of the taxonomy classification affects the quality of the final assembly.

For the five transcriptomes assembled from the RNA-Seq samples, fewer transcripts match isoforms with < 80% coverage. The SRA-based assemblies show, as reported by rnaQuast, high duplication levels. In addition, in these cases, > 5000 transcripts do not overlap any annotated transcript (Fig. 3, category “No matching annotated isoform”). These transcripts are peculiar in that they align with the genome in a single BLAST HSP that covers the entire transcript. Most also show an expression level, or TPM value, close to the cutoff used to discard lowly expressed transcripts.

**Fig. 4:**
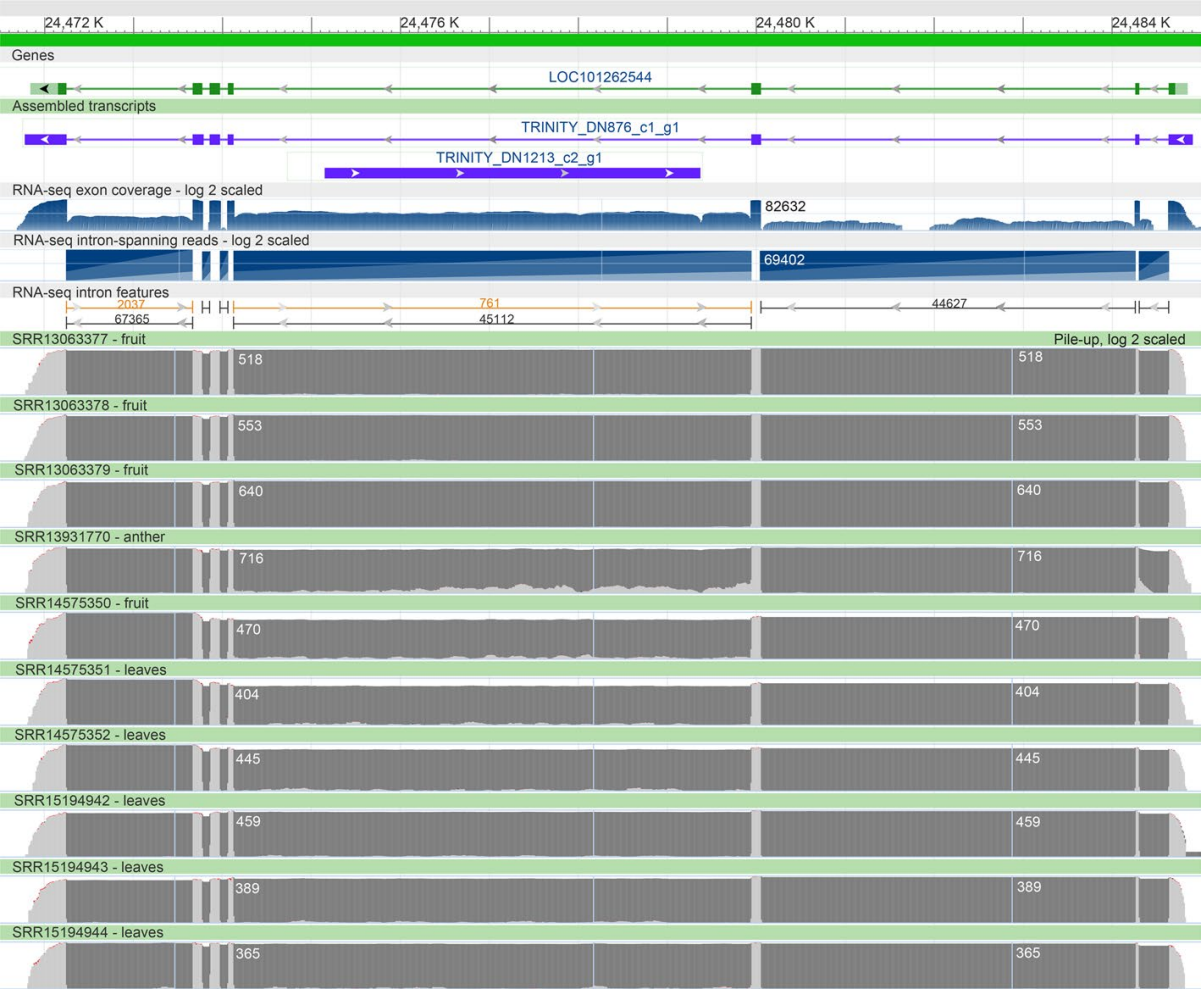
Genomic view for the annotated gene LOC101262544 (green) and aligned Trinity transcripts to the same genomic region: TRINITY_DN876_c1_g1_i2 and TRINITY_DN1213_c2_g1_i2 (purple). *Note:* Annotated exon coverage, intron spanning reads, and intro features; pile-up of alignment coverage in log_2_ scale for the SRA samples used in this study

Figure 4 shows an example in a genomic context, where one of the unannotated transcripts (TRINITY_DN1213_c2_g1_i2) does not match any annotated isoform, and is aligned with an intron of TRINITY_DN876_c1_g1_i2 (in purple) in the same genomic region. The first one matched exactly to the annotated gene, LOC101262544. It is clearly validated by the RNA-Seq alignment coverage and the spanning reads (dark grey gaps between exons) in all SRA samples in this study. Samples SRR13931770 and SRR14575350 collected from the anther and fruit tissues, respectively, however, show some intronic sequence coverage that was used by Trinity to assemble TRINITY_DN1213_c2_g1_i2. We should clarify that these two SRA samples belong to different BioProjects and were collected independently. It is difficult to determine whether this is DNA contamination or an artifact of the experimental assembly protocol. In our opinion, this is a false-positive transcript that should be eliminated from the final assembly. It is difficult or practically impossible, however, to detect this class of false positives when the target organism does not have a reference genome.

**Fig. 5:**
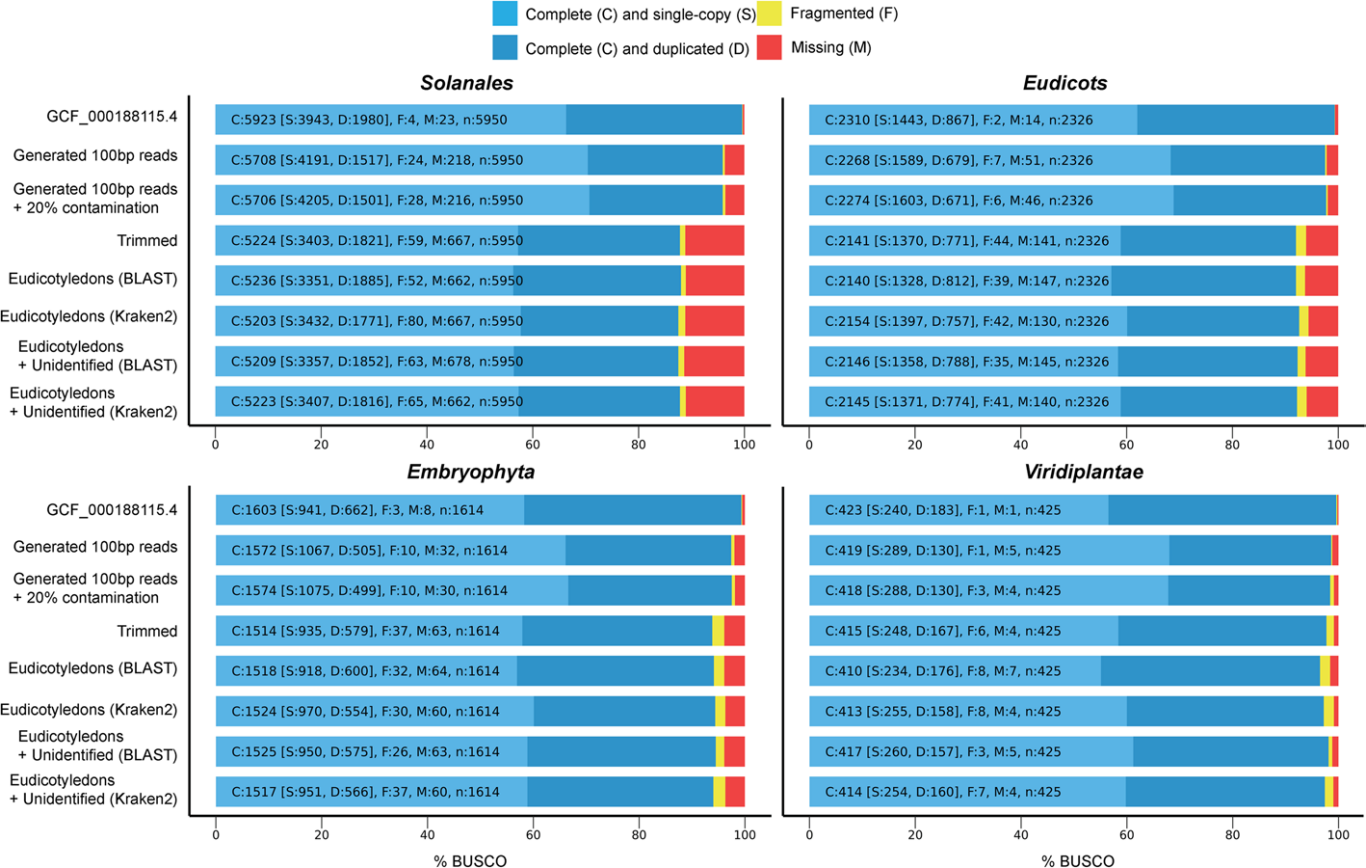
BUSCO profiles for the reference transcriptome and the seven assembled transcriptomes generated at different taxonomic levels

We also performed BUSCO analyses to assess the completeness of the assemblies. BUSCO profile plots were generated for four taxonomic levels to compare the reference and the seven de novo assembled transcriptomes (Fig. 5). The profile plot created for the *Solanales* order shows that the 100-bp generated assemblies are similar, with a difference of only two missing BUSCO profiles. The same differences are present when comparing the assemblies generated from the SRA samples. Although the Eudicotyledons assembly is less fragmented and is missing some BUSCO profiles, there is no significant difference in comparison to the other assemblies with respect to the conserved BUSCO profiles. The same pattern is present in the BUSCO plots for the *Eudicots* and *Embryophyta* clades. The Eudicotyledons assembly, however, is more fragmented and is missing BUSCO profiles in the *Viridiplantae* kingdom compared with the other two SRA-based assemblies.

The BUSCO plots demonstrate that RNA-Seq contamination does not affect the highly conserved genes at different taxonomic levels. Further, our decontamination steps, which remove contaminant and chimeric transcripts, do not affect the BUSCO completeness of the assembly as well.

After annotation of the assembled transcripts, the transcriptome assembly process is completed. Assembled transcripts can be annotated and cross-referenced with public databases, such as GO [10], NCBI Conserved Domain Database [51], COG [52], Pfam [53], UniProt [54], and eggNOT [55]. Trinotate [56], for instance, is a popular transcriptome assembly and annotation framework that uses Trinity for the assembly and most of the aforementioned databases for the annotation. This reduces the possibility of reporting chimeric transcripts as relevant biological entities. We also recommend prioritizing annotated transcripts when using de novo transcriptomes as a reference in differential gene expression analyses.

## Conclusions

WTS is a valuable technology to study a wide range of biological processes even if the target organism does not have a reference genome. In this case, de novo transcriptome assemblers, such as Trinity, can be used to produce reference transcriptomes with a high level of assembly completeness and specificity. These tools, however, generate some fragmented and chimeric transcripts that are difficult to identify without a reference genome. In our opinion, an assembly is completed when the transcripts are annotated and cross-referenced to public databases. These annotations can be used as extra validations of assembled transcripts.

Foreign and cognate RNA-Seq contamination removal is a critical step in the assembly process. Although it is not included in most popular assembly pipelines, RNA-Seq contamination, if not removed, increases the number of chimeric transcripts, which affects downstream analysis. We recommend the use of GTax, a taxonomic structured database of genomic sequences, for detecting foreign contamination of transcriptome and genome sequencing data. Although we tested GTax with BLASTN and Kraken2, the database also can be searched with other tools to accelerate computation. A python package to generate a GTax database from the NCBI Genome database is available at https://gtax.readthedocs.io/.

Transcriptome assembly is a complex process that requires the integration of many bioinformatics tools and methodologies usually in a pipeline. The assembler is not the only critical step; pre-processing steps to prepare the data and post-processing steps, such as vector detection, contamination removal, and final annotation, make a de novo assembly a viable transcriptome reference for further analysis.

## Methods

### GTax

Assembly metadata for four taxonomy superkingdoms (*Archaea*, *Bacteria*, *Viruses*, and *Eukaryotes*) were gathered using NCBI Datasets, version 12.19.0. Only RefSeq genomic sequences were used because, of the three main genomic data host institutes, NCBI is the only one that uses a contamination screening pipeline for WGS data submissions. Each superkingdom set of metadata was processed with an *in-house* developed and freely available python package (https://gtax.readthedocs.io/). The first step of the filtering process is to select, for each taxonomy, the reference genome, if available, or the latest assembly. Then, unplaced sequences inside the assemblies are discarded because most include contamination. Finally, sequence accessions starting with RefSeq prefixes such as NW and NZ were excluded, except for the case of NZ_CM and NZ_CP, which are the codes for complete chromosomes in GenBank. GTax taxonomy groups were created with three files: FASTA, text file with the relationship between sequence accession and TaxID (used to create the BLAST databases with taxonomy information), and a final file with the same relationship plus the file offset where the sequence can be extracted directly.

### RNA-Seq processing

We used standalone BLAST version 2.13.0+ to identify matches between the reads and GTax sequences. BLAST parameters used to define a match were (a) percentage of identity larger than 75%, (b) query (read) coverage larger than 75%, (c) *e*-value smaller than 1.0 × 10^−5^, and (d) the penalty for nucleotide mismatch equals −3. FASTQ files were transformed to FASTA and divided into files that contained 50,000 sequences each to speed up processing.

Kraken2 version 2.1.2 with default options was also used to identify matches between the reads and GTax sequences.

### Assemblies

Trinity version 2.13.2 with default parameters was used to generate the assemblies. Transcript quantification was executed as described in the manual (http://trinityrnaseq.github.io/) using script: align_and_estimate_abundance.pl and abundance estimation method Kallisto. A TPM cutoff of 2.5 was used to filter out lowly expressed transcripts. BUSCO version 4.1.2 with databases odb10 was used to generate the BUSCO profiles, using default parameters. RNAQuast version 2.2.1 with default parameters was used to compare the assemblies generated in this study.

### Supplementary Information

**Additional file 1. **Datasets and metadata for the creation of GTax (see Gtax 2021 tab).

**Additional file 2: Supplementary Table 3A.** Samples from SRA database for organisms without a reference genome processed with Kraken2. **Supplementary Table 3B.** Samples from SRA database for organisms without a reference genome processed with Kraken2.

**Additional file 3: Table 1.** Tomato WT samples. **Table 2.** rnaQuast short report. **Table 3.** annot. Vs ssemb.

**Additional file 4.** Review history.

## Data Availability

GTax [57] is implemented as a Python package under Public Domain license. Source code is available at https://github.com/ncbi/gtax [58] and documentation is available at https://gtax.readthedocs.io/. The current version of GTax FASTA files is available for download at: https://console.cloud.google.com/storage/browser/gtax-database. All RNA-Seq data used in this study are publicly available on the NCBI Sequence Read Archive (https://www.ncbi.nlm.nih.gov/sra). RNA-Seq samples used to validate GTax are SRR5823570 [59], SRR16571653 [60], SRR3340606 [61], SRR1952996 [62], SRR3478177 [63], SRR5178307 [64], SRR3084452 [65], SRR3297746 [66], SRR8242436 [67], SRR16661141 [68], SRR12442784 [69], SRR16958449 [70], SRR14160197 [71], SRR4249968 [72], SRR9160217 [73], DRR185733 [74], and SRR7734450 [75]. Tomato RNA-Seq samples used to study de novo transcriptome assembly are SRR13063378 [76], SRR13063378 [77], SRR13063379 [78], SRR13931770 [79], SRR14575350 [80], SRR14575351 [81], SRR14575352 [82], SRR15194942 [83], SRR15194943 [84], and SRR15194944 [85].
