# Supplementary material for: GTax: improving de novo transcriptome assembly by removing foreign RNA contamination
Source: Genome Biol. 2024 Jan 8;25:12. doi: 10.1186/s13059-023-03141-2 (PMC10773103; doi:10.1186/s13059-023-03141-2)
Supplement: Supplementary file 2 — Additional file 2: Supplementary Table 3A. Samples from SRA database for organisms without a reference genome processed with Kraken2. Supplementary Table 3B. Samples from SRA database for organisms without a reference genome processed with Kraken2. [file 13059_2023_3141_MOESM2_ESM.docx]

Supplementary Table 3A: Samples from SRA database for organisms without a reference genome processed with Kraken2.

| Organism | Pseudomonas fluorescens | Cylindrospermopsis raciborskii FACHB-1096 | Lolium perenne | Physalis peruviana | Opuntia streptacantha | Diplocarpon rosae | Cimex lectularius | Synodus sp. isolate FZ12 FC-2018 |
| --- | --- | --- | --- | --- | --- | --- | --- | --- |
| Sample | SRR5823570 | SRR16571653 | SRR3340606 | SRR1952996 | SRR3478177 | SRR5178307 | SRR3297746 | SRR8242436 |
| Taxonomy group in GTax | Bacteria | Bacteria | Liliopsida | Eudicotyledons | Eudicotyledons | Fungi | Arthropoda | Neoteleostei |
| Reads | 2,302,977 | 12,431,662 | 11,062,381 | 21,845,419 | 41,904,616 | 658,128 | 8,527,345 | 6,811,855 |
| Reads to assembly | 99.92 | 98.52 | 98.88 | 97.93 | 93.79 | 98.26 | 97.14 | 80.64 |
| Unidentified | 2.42 | 39.74 | 88.98 | 53.27 | 67.20 | 48.51 | 87.72 | 51.61 |
| Bacteria | 97.50 | 58.77 | 0.05 | 0.49 | 2.90 | 0.47 | 0.17 | 3.12 |
| Archaea | 0.00 | 0.00 | 0.00 | 0.00 | 0.00 | 0.01 | 0.02 | 0.01 |
| Liliopsida | 0.00 | 0.09 | 9.90 | 0.12 | 1.13 | 0.13 | 0.06 | 0.32 |
| Eudicotyledons | 0.02 | 0.15 | 0.06 | 44.66 | 26.59 | 0.24 | 0.29 | 6.78 |
| Viridiplantae | 0.00 | 0.01 | 0.00 | 0.06 | 0.36 | 0.03 | 0.01 | 0.06 |
| Fungi | 0.00 | 0.55 | 0.01 | 1.01 | 0.03 | 49.75 | 0.04 | 0.04 |
| Arthropoda | 0.00 | 0.08 | 0.06 | 0.09 | 0.16 | 0.16 | 9.42 | 1.11 |
| Neoteleostei | 0.01 | 0.12 | 0.06 | 0.07 | 0.30 | 0.16 | 0.26 | 29.04 |
| Actinopterygii | 0.01 | 0.07 | 0.06 | 0.03 | 0.18 | 0.10 | 0.18 | 3.01 |
| Glires | 0.00 | 0.11 | 0.14 | 0.03 | 0.22 | 0.06 | 1.08 | 0.83 |
| Primates | 0.01 | 0.14 | 0.28 | 0.01 | 0.18 | 0.03 | 0.21 | 0.35 |
| Carnivora | 0.01 | 0.02 | 0.01 | 0.02 | 0.11 | 0.05 | 0.12 | 0.48 |
| Artiodactyla | 0.00 | 0.03 | 0.01 | 0.02 | 0.09 | 0.04 | 0.09 | 0.44 |
| Amphibia | 0.00 | 0.02 | 0.01 | 0.01 | 0.10 | 0.03 | 0.03 | 0.37 |
| Sauropsida | 0.01 | 0.06 | 0.03 | 0.03 | 0.19 | 0.10 | 0.11 | 1.04 |
| Sarcopterygii | 0.00 | 0.03 | 0.02 | 0.04 | 0.16 | 0.06 | 0.11 | 0.82 |
| Chordata | 0.00 | 0.00 | 0.00 | 0.00 | 0.04 | 0.03 | 0.01 | 0.26 |
| Eukaryota | 0.00 | 0.01 | 0.01 | 0.02 | 0.04 | 0.04 | 0.05 | 0.16 |
| Viruses | 0.00 | 0.00 | 0.30 | 0.00 | 0.01 | 0.01 | 0.00 | 0.15 |

*Note.* Values expressed in percentages. Reads in cell of the same color for each sample are summed to generate the final reads to assembly.

Supplementary Table 3B: Samples from SRA database for organisms without a reference genome processed with Kraken2.

| Organism | Acipenser sp | Cavia porcellus | Homo sapiens | Ursus americanus | Eptesicus fuscus | Spea bombifrons | Taeniopygia guttata | Influenza A virus |
| --- | --- | --- | --- | --- | --- | --- | --- | --- |
| Sample | SRR16661141 | SRR12442784 | SRR16958449 | SRR14160197 | SRR4249968 | SRR9160217 | DRR185733 | SRR7734450 |
| Taxonomy group in GTax | Actinopterygii | Glires | Primates | Carnivora | Artiodactyla | Amphibia | Sauropsida | Viruses |
| Reads | 21,742,680 | 24,000,000 | 16,818,866 | 17,092,718 | 25,227,832 | 21,336,768 | 17,778,851 | 9,731,528 |
| Reads to assembly | 99.13 | 99.33 | 21.91 | 96.56 | 83.65 | 88.47 | 99.85 | 18.96 |
| Unidentified | 4.66 | 3.06 | 0.66 | 1.41 | 48.87 | 76.28 | 0.75 | 8.18 |
| Bacteria | 0.06 | 0.38 | 77.88 | 0.13 | 0.09 | 3.00 | 0.02 | 1.99 |
| Archaea | 0.00 | 0.00 | 0.00 | 0.00 | 0.00 | 0.00 | 0.00 | 0.00 |
| Liliopsida | 0.02 | 0.01 | 0.01 | 0.01 | 0.06 | 0.58 | 0.01 | 0.99 |
| Eudicotyledons | 0.03 | 0.01 | 0.03 | 1.13 | 0.08 | 2.12 | 0.02 | 0.29 |
| Viridiplantae | 0.00 | 0.00 | 0.00 | 0.00 | 0.02 | 0.00 | 0.00 | 0.01 |
| Fungi | 0.00 | 0.00 | 0.00 | 0.00 | 0.02 | 0.04 | 0.00 | 0.01 |
| Arthropoda | 0.03 | 0.02 | 0.02 | 0.16 | 0.20 | 0.19 | 0.01 | 0.58 |
| Neoteleostei | 0.29 | 0.03 | 0.03 | 0.08 | 0.85 | 1.73 | 0.03 | 1.45 |
| Actinopterygii | 94.47 | 0.01 | 0.01 | 0.88 | 0.47 | 1.73 | 0.01 | 0.70 |
| Glires | 0.09 | 96.27 | 0.02 | 0.04 | 3.49 | 0.40 | 0.01 | 9.96 |
| Primates | 0.02 | 0.09 | 21.25 | 0.19 | 2.50 | 0.12 | 0.01 | 18.54 |
| Carnivora | 0.01 | 0.04 | 0.01 | 95.15 | 3.33 | 0.08 | 0.01 | 46.41 |
| Artiodactyla | 0.01 | 0.03 | 0.01 | 0.64 | 34.78 | 0.13 | 0.01 | 0.04 |
| Amphibia | 0.01 | 0.00 | 0.01 | 0.00 | 0.10 | 12.19 | 0.00 | 0.01 |
| Sauropsida | 0.24 | 0.01 | 0.03 | 0.02 | 0.26 | 0.56 | 99.10 | 0.02 |
| Sarcopterygii | 0.02 | 0.03 | 0.02 | 0.03 | 4.79 | 0.77 | 0.01 | 0.02 |
| Chordata | 0.01 | 0.00 | 0.01 | 0.00 | 0.02 | 0.06 | 0.00 | 0.00 |
| Eukaryota | 0.00 | 0.00 | 0.00 | 0.00 | 0.01 | 0.02 | 0.00 | 0.00 |
| Viruses | 0.00 | 0.00 | 0.00 | 0.13 | 0.04 | 0.00 | 0.00 | 10.78 |

*Note.* Values expressed in percentages. Reads in cell of the same color for each sample are summed to generate the final reads to assembly.
