# Supplementary material for: GTax: improving de novo transcriptome assembly by removing foreign RNA contamination
Source: Genome Biol. 2024 Jan 8;25:12. doi: 10.1186/s13059-023-03141-2 (PMC10773103; doi:10.1186/s13059-023-03141-2)
Supplement: Supplementary file 4 — Additional file 4. Review history. [file 13059_2023_3141_MOESM4_ESM.docx]

**1^st^ round**

**Reviewer 1**

This article presents a bioinformatic study of the contamination issue in transcriptomics. The authors pointed out that the lack of reference genomes of some organisms is a source of contamination. To remove those contaminants, they developed GTax database, which contains reference genomes organized in individual taxonomic groups. Using this database with BLAST, a user can identify whether individual sequencing reads are classified to the correct taxonomic group, or to wrong ones, the latter of which indicate contamination. They tested this workflow using some simulated and real transcriptomic datasets, and found it effective.
While this work could potentially be useful to the field of transcriptomics, I have multiple concerns, as detailed below.
GTax is basically a truncated NCBI database that contain a subset of the sequences as compared with the larger nt and nr. While the article made a valid point of the logic of creating GTax (nt and nr are too large, page 8 line 16), it doesn't provide more justification for developing another NCBI database. If I understand correctly, RefSeq assemblies under certain taxonomy group can be easily retrieved by filtering the assembly report provided in the NCBI FTP server. This step is easy enough for users with average bioinformatics skills. In fact, many users do customize their databases. I do not see that the design of GTax (page 8) has more sophisticated considerations that averages users cannot achieve.
The tradeoff between database size and alignment accuracy is underexplored in this article. It simply states "Because BLAST remains the most sensitive tool for sequence similarity searches" (page 8, line 19). However, it is year 2022, and there is a big list of faster alignment or taxonomic classification tools: Salmon, Kallisto, Kraken2, Bowtie2, Minimap2, Vsearch, Diamond, on and on. They might be less accurate than BLAST (but not much, as shown in many studies), but they could work with much larger databases, and this gain of sensitivity might compensate for the (slight) reduction of precision. I understand that the authors are affiliated with NCBI and they may be most familiar with NCBI's famous BLAST. But ignoring all other tools is not a good justification for developing GTax.
The strategies for labeling a sequencing read as correct or contaminant (page 9) sounds bit less rigorous to me. It appears that a read is labeled as contaminant only when it can be aligned to a certain taxonomic group that does not the target organism. That is, the user must know the potential source of contamination before they perform this analysis. This is not only inefficient, but also introduces human bias. On the other side, reads are labeled as correct only when they can be aligned to a target taxonomic group. What if - the sequenced genome has some regions that are not present in any of the reference genomes? These regions could be of biological interest as they represent novel genetic materials, as compared with references. But in the described pipeline, these sequences will be dropped, which is probably not favored.
I also find the strategies for testing the GTax + BLAST workflow (pages 3 and 10) less robust. To simulate contaminants, the authors randomly selected sequences from the GTax and added them to the input transcriptomic datasets. This method is fine, but it does not simulate the more common scenario where the contamination is from organisms whose genomes were not sequenced or included in GTax. To resolve this, a better method would be removing the reference sequences that are the sources of contamination from GTax, they test whether the truncated GTax can still identify the contamination. This logic is analogous in the training / test split in machine learning.
I would also comment on the data presentation methods in this manuscript. The most important results are presented in barplots and tables, which appear to be generated using Microsoft Office. In year 2022, there are more effective visualization methods that can both reveal the trend of data and save space .
I do not understand why "background" is under "Results and Discussion" when it could have been part of "Introduction".
There are some artifacts in the text. For example: page 8 line 43.

**Reviewer 2**

The manuscript describes the construction of GTax, a collection of genomic sequences designed for the efficient identification of contaminant short reads in RNAseq datasets with BLASTN, which still is more sensitive than K-mer approaches, at the cost of being relatively slow.

Main comments:

page5 line 16) The authors say "When no reference genome is available for the target organism this ability remains problematic" but no more context is given. My first thought was that perhaps transcripts/partial ESTs are not included in NCBI NT but then I think they are, aren't they? I can think of examples of cloned plant genes, submitted to GenBank or the ENA, that pop up in BLAST searches. In fact NR and NT are introduced later on, on page 8. I think it will help the reader if reasons to justify this statement are listed.

p8 l33) When you say "reference genomes", do you mean RefSeq genomes? Please reword here; instead it is properly explained in methods.

p8 table1) Does the column "Sequences" actually mean "genome sequences"? Please correct column name.

p9 l14) It is not clear to me the meaning of "GTax represents 72.18% of NT", how is this computed? Please explain. Also, how much is 6% of NT today, can that fit a laptop RAM?

p9 l36) This paragraph summarizes how RNAseq reads are classified by GTax. I think the protocol adequately tackles the defined problem and the authors already acknowledge the limitation that it won't classify any contaminants from sources not in the NCBI. However, I feel it still lacks a key point: what would happen in challenging situations, such as species with large fractions of HGT, are contaminants ever mixed up in this setting?

p9 l50) Please explain if these BLAST searches can be only performed online at the NCBI servers or also from locally installed BLAST binaries. In fact, it would help if table 1 contained data on how long/how much RAM do these jobs require if run locally. Moreover, more details are needed to replicate this searches. For instance, what were the parameters of the search, can the same read align more than once, is there a min required sequence identity for matches? Are they different to the parameters used in https://bmcbiol.biomedcentral.com/articles/10.1186/s12915-018-0486-7 ?

p10 table2) It is confussing that percentages (ie 98.53%) and individual aligned reads (340) are mixed in the same rows. In addition, I guess it would help if a row named "Unidentifed" is added so the numbers add up. I then noticed that table3 has percentages all over...

p11 l41) Please explain the rationale to call "reads for assembly" as the sum of Unidentified + proper taxon. This seems a bit confusing to me. When I look at the Lollium example, 93.59% of reads where "unidentified" even when there are several Triticeae genomes in RefSeq. My point is: if the BLASTN search cannot match those Triticeae, nor largely none of the other taxa in GTax, how can we be sure they are bona fide non-contaminants? If the search is not sensitive enough to match to other grasses, it might be equally failing to match potential contaminants? You might be right, but please explain it in simple terms. On second read I realize this point is actually illustrated on figure 2, where it can bseen that adding unidentified reads actually increases contaminants. Please comment.

p13 l24) What are GCP projects? Please explain and see also my comment p9 l50.

p14 figure 1) Please explain in detail how TPMs were computed so these steps can be reproduced.

sections describing figures 2-3) These results illustrate the effect that contaminant reads can have on assembled transcriptomes. While the reported results are interesting, I feel they are not particularly novel (see for instance https://bmcbiol.biomedcentral.com/articles/10.1186/s12915-018-0486-7) nor they help easure the performance of GTax directly (Please see my comments p9 l36 & p11 l41).

figure 4) I think your discussion of this result is reasonable, may I suggest the possibility of that transcript being an antisense RNA? Anyway, not sure this figure is that important for the paper.


Minor fixes:

p5 l6) Move the stop after the reference (2). There are other similar cases on the same page and elsewhere

p8 l41) please fix reference


Final remarks: once the limitations listed above are taken care of, I think this work has the potential to be of broad interest and utility

**Authors’ response**

Reviewer 1

Response: We thank the referee for the comments and for his time dedicated to this manuscript.

**This article presents a bioinformatic study of the contamination issue in transcriptomics. The authors pointed out that the lack of reference genomes of some organisms is a source of contamination. To remove those contaminants, they developed GTax database, which contains reference genomes organized in individual taxonomic groups. Using this database with BLAST, a user can identify whether individual sequencing reads are classified to the correct taxonomic group, or to wrong ones, the latter of which indicate contamination. They tested this workflow using some simulated and real transcriptomic datasets and found it effective.
While this work could potentially be useful to the field of transcriptomics, I have multiple concerns, as detailed below.**

**GTax is basically a truncated NCBI database that contain a subset of the sequences as compared with the larger nt and nr. While the article made a valid point of the logic of creating GTax (nt and nr are too large, page 8 line 16), it doesn't provide more justification for developing another NCBI database. If I understand correctly, RefSeq assemblies under certain taxonomy group can be easily retrieved by filtering the assembly report provided in the NCBI FTP server. This step is easy enough for users with average bioinformatics skills. In fact, many users do customize their databases. I do not see that the design of GTax (page 8) has more sophisticated considerations that averages users cannot achieve.**

Response:

We thank the reviewer for  their comments and analysis.

We disagree that GTax is just a “truncated” set of NCBI RefSeq database sequences. GTax is a taxonomically organized set of files that allows for an easy taxonomic classification of RNASeq raw read sequences. It is correct that advanced bioinformaticians could create similar subset from NCBI RefSeq flat files but, in our opinion, this requires a knowledge of the NCBI data organization and advanced programming skills to deal with the NCBI Taxonomy data tree structure. More importantly, we provide a python package that creates GTax FASTA files and TAXID maps with minimum effort and quickly . The python package is available as a module that can be used to load GTax’s python pickle objects that store the NCBI Taxonomy database in graph as it is described in this tutorial <https://gtax.readthedocs.io/en/latest/database.html>. Proof of the utility of GTax is in the results of the many experiments that we present. In addition, we clearly show that including searching the GTax database in our analysis pipeline efficiently identifies contamination in sequences.

**The tradeoff between database size and alignment accuracy is underexplored in this article.**

Response:

We processed simulated short reads from organisms with assemblies in GTax and demonstrated that more than 98% of the reads from these organisms are aligned with the correct taxonomy group (see Table 2A and B). A second evaluation was executed with organism with not genomic sequence in GTax, see Table 3A and B. In this case, for the bacterium *Pseudomonas fluorescens*, 92.35% of the reads aligned with the correct taxonomy group due to being phylogenetically close to other bacteria in the GTax group. Additionally, for other less annotated organisms like *Eptesicus fuscus*, a high percent of the reads remains unidentified. Also, % of contaminant reads are similar to those reported by the NCBI Sequence Taxonomic Analysis Tool (STAT), an internal tool that is used for detecting contamination on SRA submissions. We think we adequately addressed the alignment issue and database size. Furthermore, we do not think that our study addressed database size and alignment accuracy *per se.* That would be an extensive study for separate manuscript.

**It simply states "Because BLAST remains the most sensitive tool for sequence similarity searches" (page 8, line 19). However, it is year 2022, and there is a big list of faster alignment or taxonomic classification tools: Salmon, Kallisto, Kraken2, Bowtie2, Minimap2, Vsearch, Diamond, on and on. They might be less accurate than BLAST (but not much, as shown in many studies), but they could work with much larger databases, and this gain of sensitivity might compensate for the (slight) reduction of precision. I understand that the authors are affiliated with NCBI and they may be most familiar with NCBI's famous BLAST. But ignoring all other tools is not a good justification for developing GTax.**

Response:

NGS aligners like Salmon, Kallisto, Bowtie2, etc., are not useable for taxonomy classification as they need a reference genome for aligning sequence reads. In this study, we aim to identify contaminant reads and the source organisms are unknown. We used BLAST as it is the most sensitive available tool to achieve high quality results. However, due to BLAST consuming significant resources, it is not suitable for production environments. We tested the combination of GTax and Kraken2. See supplementary Tables S3A and S3B that show contamination detected with Kraken2 for the SRA samples analyzed in the manuscript.

As expected, Kraken2 is less stringent than BLAST, meaning that less reads remain unidentified. These are expected results as Kraken2 uses a k-mer based algorithm with maximum k-mer length of 35 which is much bigger that our read lengths (>= 100 bp). Local alignments of 35 bp can occur but this only represent 35% of the query read if the read is 100bp or less than that for longer reads. BLAST options were modified to report alignments with query coverage larger than 75% of the read which made BLAST based alignments more sensitive than those reported by Kraken2. Nevertheless, Kraken2 results are very similar to BLAST. The manuscript text was modified with this data on page 13 from line 19.

**The strategies for labeling a sequencing read as correct or contaminant (page 9) sounds bit less rigorous to me. It appears that a read is labeled as contaminant only when it can be aligned to a certain taxonomic group that does not the target organism. That is, the user must know the potential source of contamination before they perform this analysis. This is not only inefficient, but also introduces human bias.**

Response:

Our algorithm uses a two-step approach to detect contamination when sources are totally unknown. In the first step, all reads are screened to the target organism’s GTax taxonomy group. This is something users know since they know which organism they are studying (ie. where they isolated the sample from). Here, we assume that RNA-Seq reads from a target organism will be aligned to the target organism’s GTax taxonomy group when the target organism has a reference genome or an assembly at any level included in GTax; these reads are identified as “correct”, or not contaminant reads. If there are no sequences for the target organism in GTax, but genomic sequences from a phylogenetically closely related species, some percent of the reads will align with the correct taxonomy group. The special case when no similar sequences are in the target organism’s GTax taxonomy group, (ie. a totally unannotated organism), the reads will be classified as “unidentified”.

For the second step, all “unidentified” reads in the previous step are screened against the rest of GTax taxonomy groups. This step, identifies “known” contaminants, meaning that reads which align to organisms with sequences in GTax that are not in the target organism’s GTax taxonomy group are classified as “known” contaminants.

Taxonomy classification is limited to the sequences included in the database and the confidence that those sequences are free of contaminants. It is impossible with the current state of the art to detect “unknown” contaminants if there is no sequence information in the database.

**On the other side, reads are labeled as correct only when they can be aligned to a target taxonomic group. What if - the sequenced genome has some regions that are not present in any of the reference genomes? These regions could be of biological interest as they represent novel genetic materials, as compared with references. But in the described pipeline, these sequences will be dropped, which is probably not favored.**

Response:

In this special case, the reads will remain “unidentified”.. Most of them, will also not be aligned to any other GTax group and remain “unidentified” only after the second screen, see Table 3A and 3B. For this reason, we present assemblies including the “unidentified” ones but alert users that this will require extra analysis as “unknown” contaminant reads may remain as well due to the limitation of annotated data. See the discussion of Table 3A and 3B.

**I also find the strategies for testing the GTax + BLAST workflow (pages 3 and 10) less robust. To simulate contaminants, the authors randomly selected sequences from the GTax and added them to the input transcriptomic datasets. This method is fine, but it does not simulate the more common scenario where the contamination is from organisms whose genomes were not sequenced or included in GTax. To resolve this, a better method would be removing the reference sequences that are the sources of contamination from GTax, they test whether the truncated GTax can still identify the contamination. This logic is analogous in the training / test split in machine learning.**

Response:

As detailed before, detecting fully “unknown” organisms, which means an organism with no genomic sequence information is pushing the limited of this approach. Our intention is to remove all contaminants that are known and leave the “unidentified” reads as a product that need to be treated with caution. We used the SRA samples of non-included organisms in GTax for this intent, Tables 3A and 3B.

**I would also comment on the data presentation methods in this manuscript. The most important results are presented in barplots and tables, which appear to be generated using Microsoft Office. In year 2022, there are more effective visualization methods that can both reveal the trend of data and save space .**

Response:

We thank the reviewer for their suggestion. However, we prefer to keep the way we present our data in tables and bar plots. This is a simple way to explain our results that can be understood by a wide audience. We do not think that we are missing any important result or interpretation by our method.

**I do not understand why "background" is under "Results and Discussion" when it could have been part of "Introduction".
There are some artifacts in the text. For example: page 8 line 43.**

Response:

We have reorganized the manuscript sections as suggested by the reviewer.

Reviewer 2

Response: We thank the referee for the comments and for his time dedicated to this manuscript.

**The manuscript describes the construction of GTax, a collection of genomic sequences designed for the efficient identification of contaminant short reads in RNAseq datasets with BLASTN, which still is more sensitive than K-mer approaches, at the cost of being relatively slow.**

**Main comments:

page5 line 16) The authors say "When no reference genome is available for the target organism this ability remains problematic" but no more context is given. My first thought was that perhaps transcripts/partial ESTs are not included in NCBI NT but then I think they are, aren't they? I can think of examples of cloned plant genes, submitted to GenBank or the ENA, that pop up in BLAST searches. In fact NR and NT are introduced later on, on page 8. I think it will help the reader if reasons to justify this statement are listed.**

Response:

If a reference genome is available, contaminated reads can be detected by aligning the reads to the genome. A different process is required if the target organism has an incomplete genome or no genome at all.

For more clarity, this line was rephrased to: “Contamination, however, is more common than expected in RNA-Seq samples (25, 26); it is particularly problematic in samples from organisms for which there is no reference genome in which to frame the analysis (27).”

**p8 l33) When you say "reference genomes", do you mean RefSeq genomes? Please reword here; instead it is properly explained in methods.**

Response:

The author is correct, GTax is comprised of RefSeq genomes only. We reworded the sentence in the manuscript.

**p8 table1) Does the column "Sequences" actually mean "genome sequences"? Please correct column name.**

Response:

We corrected the table column to “RefSeq Sequences”. These numbers represent the number of RefSeq sequences included in each taxonomy group.

**p9 l14) It is not clear to me the meaning of "GTax represents 72.18% of NT", how is this computed? Please explain. Also, how much is 6% of NT today, can that fit a laptop RAM?**

Response:

GTax includes only RefSeq reference genomes or the latest assembly if the reference genome is not available. Therefore, for each reference genome included in GTax, only RefSeq sequences of complete chromosomes are included. If no reference genome is available, then scaffolds and contigs in RefSeq sequences are included. This reduces the number of RefSeq sequences (redundance of genomic regions) included in GTax trying to minimize the size of the database.

At the time the manuscript was submitted, 6% of NT meant BLAST indexes of 15 GB which can be used in a laptop (Add page 9 line 3). The 6% of NT example refers to the *Sauropsida* taxonomy group that is the largest group in GTax. Explaining it in another way, if the target organism is the *Sauropsida* taxonomy group, the BLAST searches will be against only 6% of the NT database (page 10 line 16).

“GTax represents 72.18% of NT” means that if we get all sequences included in GTax, they are the 72.18% of the NT sequences. The rest (27.82%) NT sequences are not included in GTax. Since this is an iterative process, the first search is against the target organism’s GTax taxonomy group which is, at most, ~6% of the NT database. At this point, most of the searched sequences are identified and there are only a small number of subsequent searches necessary against the rest of GTax.

**p9 l36) This paragraph summarizes how RNAseq reads are classified by GTax. I think the protocol adequately tackles the defined problem and the authors already acknowledge the limitation that it won't classify any contaminants from sources not in the NCBI. However, I feel it still lacks a key point: what would happen in challenging situations, such as species with large fractions of HGT, are contaminants ever mixed up in this setting?**

Response:

Our algorithm uses a two-step approach to detect contamination. In the first step, all reads are screened for the target organism’s GTax taxonomy group. Reads aligned to that group of sequences will be marked as non-contaminant. Then, in the second step reads not identified in the first step are screened against the rest of GTax taxonomy groups. Marking reads as non-contaminant in the case of HGT in the target species will depend on the availability of a reference genome or the presence of similar HGT in other phylogenetically close species (species in the same GTax taxonomy group).

There are three situations that we can visualize in the case of HGT events. First, if the target organism reference genome is in GTax, all reads, including the HTG reads, that align to the target organism’s GTax taxonomy group sequences will be marked as no contaminant. Therefore, those reads will not be screened against the other GTax taxonomy groups.  Second, if no reference genome but the HTG events are common in other phylogenetically close species in the target organism’s GTax taxonomy group, then, those HTG reads will be aligned to the target organism’s GTax taxonomy group sequences and will be marked as non-contaminant. Finally, if the HTG reads cannot be aligned to any of the sequences included in the target organism’s GTax taxonomy group then our algorithm will fail, and mark those reads as contaminants. We think that these are most likely a rare case.

**p9 l50) Please explain if these BLAST searches can be only performed online at the NCBI servers or also from locally installed BLAST binaries. In fact, it would help if table 1 contained data on how long/how much RAM do these jobs require if run locally. Moreover, more details are needed to replicate this searches. For instance, what were the parameters of the search, can the same read align more than once, is there a min required sequence identity for matches? Are they different to the parameters used in** [**https://gcc02.safelinks.protection.outlook.com/?url=https%3A%2F%2Fbmcbiol.biomedcentral.com%2Farticles%2F10.1186%2Fs12915-018-0486-7&data=05%7C01%7Croberto.veraalvarez%40nih.gov%7C9f17a4f26c344e5d424908db5ad10a1b%7C14b77578977342d58507251ca2dc2b06%7C0%7C0%7C638203626079852151%7CUnknown%7CTWFpbGZsb3d8eyJWIjoiMC4wLjAwMDAiLCJQIjoiV2luMzIiLCJBTiI6Ik1haWwiLCJXVCI6Mn0%3D%7C3000%7C%7C%7C&sdata=nLUmxxJYP5EGl5CaHw9QjLNKzRATQHUNHG2LgMWCgeQ%3D&reserved=0**](https://gcc02.safelinks.protection.outlook.com/?url=https%3A%2F%2Fbmcbiol.biomedcentral.com%2Farticles%2F10.1186%2Fs12915-018-0486-7&data=05%7C01%7Croberto.veraalvarez%40nih.gov%7C9f17a4f26c344e5d424908db5ad10a1b%7C14b77578977342d58507251ca2dc2b06%7C0%7C0%7C638203626079852151%7CUnknown%7CTWFpbGZsb3d8eyJWIjoiMC4wLjAwMDAiLCJQIjoiV2luMzIiLCJBTiI6Ik1haWwiLCJXVCI6Mn0%3D%7C3000%7C%7C%7C&sdata=nLUmxxJYP5EGl5CaHw9QjLNKzRATQHUNHG2LgMWCgeQ%3D&reserved=0) ?

Response:

BLAST searches were executed using standalone BLAST version 2.13.0+ on the NIH HPC Biowulf cluster (<http://hpc.nih.gov>). BLAST parameters are described in the Methods section as (a) percentage of identity larger than 75%, (b) query (read) coverage larger than 75%, (c) e-value smaller than 1.0x10^-5^, and (d) the penalty for nucleotide mismatch equals -3. Users can rerun the analysis in their own compute environment or on the cloud. GTax sequences used on this manuscript are in <https://console.cloud.google.com/storage/browser/gtax-database> version 20211025.

Run time will depend on the number of reads to screen and the taxonomy group used as the BLAST database. In the case of the *Sauropsida* taxonomy group, BLAST, using 16 threads (*-num_threads 16*), requires 16 GB of RAM for processing on average 303 reads per minute using an AMD EPYC 7543 CPU.

CroCo, the application mentioned by the reviewer is a different approach. It executes BLAST after assembly, therefore BLAST needs parameters for aligning large sequences (assembled transcripts).

Our analysis is pre-assembly as we demonstrate that contaminant reads can affect the assembly process by creating chimeric transcripts by mixing reads from the target organism and contaminated ones.  Our BLAST parameters are adjusted to align short reads.

**p10 table2) It is confussing that percentages (ie 98.53%) and individual aligned reads (340) are mixed in the same rows. In addition, I guess it would help if a row named “Unidentifed” is added so the numbers add up. I then noticed that table3 has percentages all over…**

Response:

We changed Tables 2A and B as suggested by the reviewer including only numbers of reads and a new row for “Unidentified reads”.

**p11 l41) Please explain the rationale to call "reads for assembly" as the sum of Unidentified + proper taxon. This seems a bit confusing to me. When I look at the Lollium example, 93.59% of reads where "unidentified" even when there are several Triticeae genomes in RefSeq. My point is: if the BLASTN search cannot match those Triticeae, nor largely none of the other taxa in GTax, how can we be sure they are bona fide non-contaminants? If the search is not sensitive enough to match to other grasses, it might be equally failing to match potential contaminants? You might be right, but please explain it in simple terms. On second read I realize this point is actually illustrated on figure 2, where it can bseen that adding unidentified reads actually increases contaminants. Please comment.**

Response:

The reviewer’s analysis is very interesting and illustrates the limitations of these kinds of algorithms. Detecting RNASeq contamination is limited to the sequences included in the database with confidence that those sequences are free of contaminants. This is the main reason why we use only RefSeq sequences. NCBI is the only one of the three main genomic data host institutes that uses a contamination screening pipeline for WGS data submissions, therefore, NCBI RefSeq sequences are less prone to contamination than the other sources.

Our algorithm, in the extreme cases where the target organism neither has a reference genome nor a phylogenetically closely related organism in GTax, and most of the reads are unidentified, assumes that those unidentified reads are not contaminants. The algorithm only removes “known” contamination (reads aligned to other GTax taxonomy groups) and includes all the “unidentified” reads to be included in the assembly. In cases like this, we recommend using approaches like the one described in the CroCo paper to determine the quality of an assembly.

As shown in Figure 2, in cases of well annotated organisms, we recommend leaving the unidentified reads out of the assembly as they can increase the fragmentation of the assembled transcripts.

The GTax database used in this study was created in January 2021. It included only sequences from the reference genome *Triticum dicoccoides (wild emmer wheat)* TaxID: 85692. Checking *Triticeae* tribe now (07/19/2023) on the NCBI Datasets portal and selecting only genomes annotated by NCBI RefSeq will show only five annotated genomes with three of them added after Apr 2021. <https://www.ncbi.nlm.nih.gov/datasets/genome/?taxon=147389&annotated_only=true&refseq_annotation=true>

**p13 l24) What are GCP projects? Please explain and see also my comment p9 l50.**

Response:

GCP is Google Cloud Platform, a private cloud computing provider. To interact with this platform, users need to create Projects and associate a billing account.

<https://cloud.google.com/resource-manager/docs/creating-managing-projects>

An active GCP project is required to download GTax database from <https://console.cloud.google.com/storage/browser/gtax-database>

Users can also create a GTax database locally as described here:

<https://gtax.readthedocs.io/en/latest/datasets.html>

**p14 figure 1) Please explain in detail how TPMs were computed so these steps can be reproduced.**

Response:

Transcripts per million (TPM) are calculated using the Trinity script: **align_and_estimate_abundance.pl** as described in: <https://github.com/trinityrnaseq/trinityrnaseq/wiki/Trinity-Transcript-Quantification> with alignment method Kallisto. Added to page 22 line 8.

**sections describing figures 2-3) These results illustrate the effect that contaminant reads can have on assembled transcriptomes. While the reported results are interesting, I feel they are not particularly novel (see for instance** [**https://gcc02.safelinks.protection.outlook.com/?url=https%3A%2F%2Fbmcbiol.biomedcentral.com%2Farticles%2F10.1186%2Fs12915-018-0486-7&data=05%7C01%7Croberto.veraalvarez%40nih.gov%7C9f17a4f26c344e5d424908db5ad10a1b%7C14b77578977342d58507251ca2dc2b06%7C0%7C0%7C638203626079852151%7CUnknown%7CTWFpbGZsb3d8eyJWIjoiMC4wLjAwMDAiLCJQIjoiV2luMzIiLCJBTiI6Ik1haWwiLCJXVCI6Mn0%3D%7C3000%7C%7C%7C&sdata=nLUmxxJYP5EGl5CaHw9QjLNKzRATQHUNHG2LgMWCgeQ%3D&reserved=0)**](https://gcc02.safelinks.protection.outlook.com/?url=https%3A%2F%2Fbmcbiol.biomedcentral.com%2Farticles%2F10.1186%2Fs12915-018-0486-7&data=05%7C01%7Croberto.veraalvarez%40nih.gov%7C9f17a4f26c344e5d424908db5ad10a1b%7C14b77578977342d58507251ca2dc2b06%7C0%7C0%7C638203626079852151%7CUnknown%7CTWFpbGZsb3d8eyJWIjoiMC4wLjAwMDAiLCJQIjoiV2luMzIiLCJBTiI6Ik1haWwiLCJXVCI6Mn0%3D%7C3000%7C%7C%7C&sdata=nLUmxxJYP5EGl5CaHw9QjLNKzRATQHUNHG2LgMWCgeQ%3D&reserved=0)) **nor they help easure the performance of GTax directly (Please see my comments p9 l36 & p11 l41).**

Response:

CroCo approach identifies contamination after assembly is executed. We demonstrate in our manuscript that contaminant reads can create misconnected fragments with the target organism reads and creates several fragmented transcripts and/or chimeric transcripts that affect the quality of the assembly. This is why we recommend removing contaminated reads prior to the assembly.

**figure 4) I think your discussion of this result is reasonable, may I suggest the possibility of that transcript being an antisense RNA? Anyway, not sure this figure is that important for the paper.**

Response:

We thank the reviewer for the suggestion. We think that this example is important to illustrate that even after the many quality control steps during the assembly process, there remain a small number of false positive transcripts in the assemblies and other algorithms could be used to detect them.

**Minor fixes:**

**p5 l6) Move the stop after the reference (2). There are other similar cases on the same page and elsewhere**

Response:

Fixed in the manuscript.

**p8 l41) please fix reference**

Response:

Fixed in the manuscript.

**Final remarks: once the limitations listed above are taken care of, I think this work has the potential to be of broad interest and utility**

Response:

We thank the reviewers for the time they dedicated to our manuscript.

**2^nd^ round**

**Reviewer 1**

The authors have revised the manuscript and addressed my concerns. I see improvement of the manuscript's quality.

However, my remaining major concern is with the overall value of GTax as a bioinformatics resource (database / pipeline), especially with the assumption that it may be integrated into the NCBI platform as a "standard" resource. I generally disagree with the authors' disagreement with my concern on GTax' value. It is true that having a pre-selected set of organisms and taxonomy, and some Python code, can facilitate non-technical users (I agree with this point), especially when it is to serve the audience of NCBI. However, scientifically, I am not convinced that the presented database and pipeline represent a major advancement in the methodology of RNA-seq data analysis.

The authors attempted to address my question on "the tradeoff between database size and alignment accuracy", which I appreciate. However, I think that the authors' response does not directly address my concern. To reiterate, that I suspect that one can use a cheaper tool (like Kraken2, see below) and a larger database with the same amount of computational resource to achieve equally good or even better results. This has not been tested (see also below).

I appreciate that the authors added Kraken2 to the test, then compared and discussed the results especially regarding to the change of sensitivity. This is expected, and demonstrated the value of accurate sequence alignment (like BLAST) in contamination removal. However, the authors found that "Kraken2 results are similar to those for BLAST." We know that Kraken2 is much faster than BLAST. Given this, especially considering that the database is the same, I wonder why would a user choose BLAST over Kraken2 in this task? This question echos my previous concern. I think that the authors need to provide proper justification on this to convince the users.

As a side note, the authors' statement "NGS aligners like Salmon, Kallisto, Bowtie2, etc., are not useable for taxonomy classification as they need a reference genome for aligning sequence reads." may not be true. Bowtie2 is used under the hood by the taxonomic classifier MetaPhlAn on marker gene sequences. Kallisto was repurposed as Metakallisto for analyzing metagenomic data. Aligners like DIAMOND and MMseq2 have taxonomic classification functionality built-in, based on high-score hits.

I appreciate the authors' discussion of present and phylogenetically close organisms in the database in response to my question on the potential bias of the pipeline. I also appreciated the pipeline's treatment of "unidentified" sequences in response to my opinion that they could be useful.

**Reviewer 2**

The authors have responded to all of my previous suggestions/comments and I am satisfied with most of them.
I still have a couple of comments though, I mark with >> my previous comments and with > the responses:

> This paragraph summarizes how RNAseq reads are classified by GTax. I think the protocol
adequately tackles the defined problem and the authors already acknowledge the limitation that it
won't classify any contaminants from sources not in the NCBI. However, I feel it still lacks a key point:
what would happen in challenging situations, such as species with large fractions of HGT, are
contaminants ever mixed up in this setting?

>> Response:
Our algorithm uses a two-step approach to detect contamination. In the first step, all reads are screened
for the target organism’s GTax taxonomy group. Reads aligned to that group of sequences will be marked
as non-contaminant. Then, in the second step reads not identified in the first step are screened against
the rest of GTax taxonomy groups. Marking reads as non-contaminant in the case of HGT in the target
species will depend on the availability of a reference genome or the presence of similar HGT in other
phylogenetically close species (species in the same GTax taxonomy group).
There are three situations that we can visualize in the case of HGT events. First, if the target organism
reference genome is in GTax, all reads, including the HTG reads, that align to the target organism’s GTax
taxonomy group sequences will be marked as no contaminant. Therefore, those reads will not be
screened against the other GTax taxonomy groups. Second, if no reference genome but the HTG events
are common in other phylogenetically close species in the target organism’s GTax taxonomy group, then,
those HTG reads will be aligned to the target organism’s GTax taxonomy group sequences and will be
marked as non-contaminant. Finally, if the HTG reads cannot be aligned to any of the sequences included
in the target organism’s GTax taxonomy group then our algorithm will fail, and mark those reads as
contaminants. We think that these are most likely a rare cas

Please add these points to the discussion.
